# Tibial subchondral trabecular bone micromechanical and microarchitectural properties are affected by alignment and osteoarthritis stage

**DOI:** 10.1038/s41598-020-60464-x

**Published:** 2020-03-04

**Authors:** Jean-Baptiste Renault, Maximiliano Carmona, Chris Tzioupis, Matthieu Ollivier, Jean-Noël Argenson, Sébastien Parratte, Patrick Chabrand

**Affiliations:** 1 Aix Marseille Univ, CNRS, ISM, Marseille, France; 2 APHM, Institute for Locomotion, Department of orthopaedics and Traumatology, Sainte-Marguerite Hospital, 13009 Marseille, France; 3 Department of Orthopaedics and Traumatology, Pontificia Universidad Católica de Chile, Santiago, Chile; 4 International Knee And Joint Center, Abu Dhabi, United Arab Emirates

**Keywords:** Tissue engineering, Bone, Osteoarthritis

## Abstract

At advanced knee osteoarthritis (OA) stages subchondral trabecular bone (STB) is altered. Lower limb alignment plays a role in OA progression and modify the macroscopic loading of the medial and lateral condyles of the tibial plateau. How the properties of the STB relate to alignment and OA stage is not well defined. OA stage (KL scores 2–4) and alignment (HKA from 17° Varus to 8° Valgus) of 30 patients were measured and their tibial plateau were collected after total knee arthroplasty. STB tissue elastic modulus, bone volume fraction (BV/TV) and trabecula thickness (Tb.Th) were evaluated with nanoindentation and µCT scans (8.1 µm voxel-size) of medial and lateral samples of each plateau. HKA and KL scores were statistically significantly associated with STB elastic modulus, BV/TV and Tb.Th. Medial to lateral BV/TV ratio correlated with HKA angle (R = −0.53, p = 0.016), revealing a higher ratio for varus than valgus subjects. STB properties showed lower values for KL stage 4 patients. Tissue elastic modulus ratios and BV.TV ratios were strongly correlated (R = 0.81, p < 0.001). Results showed that both micromechanical and microarchitectural properties of STB are affected by macroscopic loading at late stage knee OA. For the first time, a strong association between tissue stiffness and quantity of OA STB was demonstrated.

## Introduction

Knee osteoarthritis (OA) is a multi-factorial disease that affects all the joint tissues and leads to significant functional disabilities. Lower limb malalignment predicts OA progression^1–4^ and is associated with load distribution within the knee joint^5^: the lateral compartment is overloaded in patients with a *valgus* deformity, and the medial compartment in patients with a *varus* deformity^6^.

The proximal tibia subchondral bone is altered by OA^7^. At the macroscopic level, as the disease progresses, the overloaded side displays increased areal bone mineral density (aBMD), revealed by macroscopic-scale measurements performed using dual-energy x-ray absorptiometry (DEXA)^4^. Moreover, bone marrow lesions, identified via magnetic resonance imaging (MRI), are found within the OA subchondral bone^8–10^, and evolve to cystlike subchondral bone lesions^11^. Recently, *in vitro* X-Ray micro-computed tomography (μCT) has allowed the investigation of the subchondral trabecular bone (STB) microarchitecture on selected volumes of interest (VOI). When applied to the subchondral bone of the human proximal tibia, μCT showed a significant regional heterogeneity in microarchitecture^12^, revealing a link between microarchitecture and degradation of the overlying cartilage^13,14^ and OA score^14^. Other studies have found differences in microarchitectural parameters between the medial and lateral subchondral regions^15,16^, which have been linked to static knee alignment^17^.

From a mechanical point of view, there is a consensus that subchondral bone tissue micromechanical properties are negatively affected in OA patients. In late-stage OA, bone is less stiff at a fixed apparent bone volume fraction^18–21^. This is explained in part by a reduced calcium/collagen ratio^22^ and hypomineralisation of the trabecular material^14,23^. The material alterations observed are consistent with the observed lower mechanical properties at microscale, as quantified by nanoindentation^24,25^.

Thus, OA affects both the micromechanical and the microarchitectural properties of bone. Moreover static knee alignment induces regional variations in microarchitectural properties in the proximal tibial bone. Where there is alteration, knowledge of how the micromechanical alterations evolve under pathology-induced modifications of microarchitecture due to macroscopic loading will contribute to a better understanding of the OA pathology. The two determinants of STB properties are microarchitecture and tissue-level mechanical properties. To the best of our knowledge, no study has investigated whether OA and static knee alignment affect STB tissue elastic properties.

Here, based on the literature outlined above, we hypothesised that knee alignment and OA stage affect both the microarchitecture of STB and its tissue-level material elastic properties. Our objectives in this study were (1) to establish whether the STB tissue modulus, determined from nanoindentation, is affected by static knee alignment and OA score, (2) to determine whether STB microarchitecture is affected by static knee alignment and OA score, (3) to evaluate the relationship between STB microarchitecture and STB tissue elastic modulus.

## Materials and Methods

### Sample collection and preparation

Thirty patients undergoing unilateral total knee arthroplasty (TKA) were included (power analysis details are in Supplementary Material). Table 1 presents their demographic and clinical data. Patients’ height and weight were recorded, and their BMI (Body Mass Index) calculated. Lower limb malalignment, quantified with reference to the hip-knee-ankle angle (HKA), was measured by the orthopedic surgeons (JNA SP MO or MC) on a bilateral weight-bearing anteroposterior radiograph of the patient’s lower limb. *Varus* alignment was defined by HKA <0° and *Valgus* by HKA> 0°. From radiographs taken in the same position and zoomed on the knee, two orthopedic surgeons (MC and CT) independently measured the Kellgren and Lawrence (KL) score of each subject twice under blinding. Patients with any bone disorders other than OA, or those who reported conditions altering bone metabolism, or currently under treatment affecting bone metabolism (e.g., anti-resorptive drugs), or receiving hormonal replacement therapy, were not included in the study. Informed consent was obtained from each subject.

**Table 1:** Patients’ demographics.

Age (yrs)	72 ± 6.1
Gender (Female: Male)	23: 7
Operated Leg (Left: Right)	11: 19
BMI	28, 6 ± 4, 8
HKA (°)	−4, 4 ± 6, 8
Knee alignment Varus: Neutral: Valgus	19: 4: 7
KL Score I: II: III: IV	0: 3: 7: 20

Demographics of the subject. Continuous variables are presented with mean ± standard deviation. For categorical variables, the numbers of each category are presented.

Each patient’s resected tibial plateau was retrieved, marked on the medial compartments for future reference and immediately placed in a jar containing a calcium buffered saline solution. The solution contained both calcium (50 mg.L^−1^ of Ca^2−^) to prevent the bone tissue mineral matrix from dissolving during conservation and sodium azide (0.01%) to avoid bacteria induced collagen degradation^26^. The samples were kept refrigerated at 4 °C. In both the medial and lateral weight-bearing regions of each plateau, a ø10 mm cylindrical core was excised using a surgical trephine bur mounted on a column drill. The drilling was done at low speed with the tibial plateau resting on its resected surface and fully immersed in the calcium buffered saline solution to avoid heating and degradation of the bone. Then, the bone thickness and the cartilage thickness of each sample was measured with a modified caliper (0.1 mm precision) along the cylindrical axis of the sample. The medial and lateral cylindrical cores were immediately placed in two different tubes, which were filled with calcium buffered saline, and refrigerated at 4 °C.

### Indentation

The samples were mounted in ABS custom cuboid sample holders to simplify their manipulation for the grinding and nanoindentation procedures. Then, the distal plane of each sample was ground on disks of successively reduced granulometry (600, 1200, 2500 grit) under constant water irrigation and polished with diamond pastes of 1 µm and 0.1 µm. Between steps, the sample was cleaned with a waterjet and via a 2-minute ultrasonic bath. Prior testing of bovine and caprine trabecular bone samples confirmed that this method yielded a sufficiently polished surface for nanoindentation. Care was taken to ensure that the post-polishing thicknesses of the subject’s medial and lateral samples were the same and that at least 0.5 mm of bone was ground to remove the regions damaged by the oscillating surgical saw^27^. After grinding, the samples were cleaned in the ultrasonic bath for 5 min, then put back in the tubes and kept refrigerated at 4 °C before the indentation phase began.

**Figure 1:**
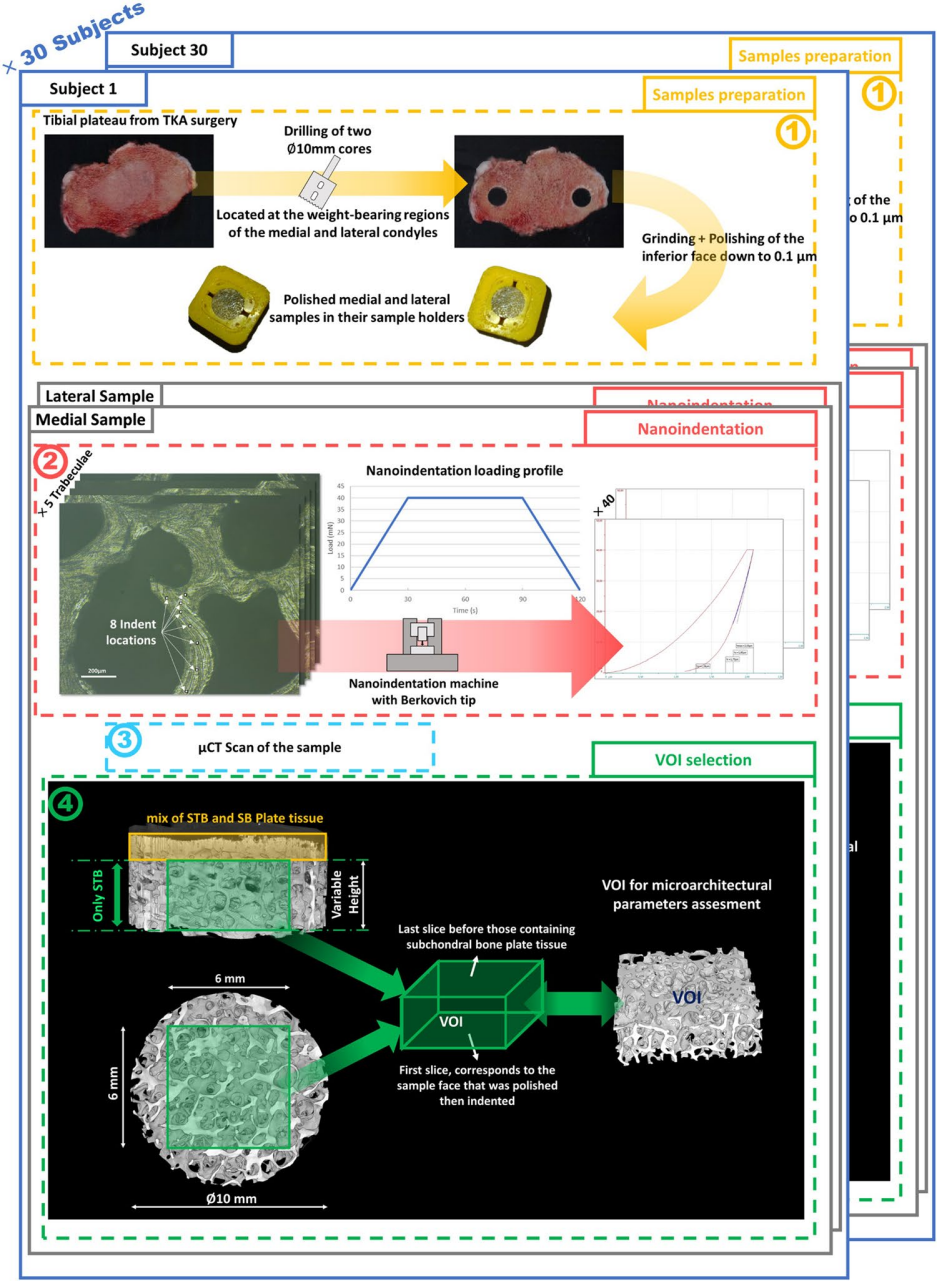
Schematic of the sample acquisitions and subsequent characterisation methods used in this study. (1) Two samples, one medial and one lateral, were excised from tibial plateaus retrieved during total knee arthroplasty surgery and polished. (2) Forty indent tests were performed per sample. (3) Each sample was μCT scanned. (4) Definition of the volume of interest (VOI) used to calculate the microarchitectural parameters.

For the indentation tests, each sample was set in a watertight support filled with the calcium-buffered saline up to the level of the polished surface. The nanoindentation apparatus (Antonn Paar, NHT²) is located in a thermally controlled room, on a pneumatic antivibration table. 40 points were selected using a x20 microscopic objective on five different trabeculae located on the polished surface of the sample. For each trabecula, 3 points were situated at the intersection with other trabeculae, while the five others were situated along the trabecula centerline to avoid any border effect. A trapezoidal loading profile (30 s: 60 s: 30 s, max load 40 mN) was applied as shown in Fig. 1. The indentation tests were performed with a Berkovich diamond tip (tip radius 60 nm). Only the distal (polished) face of each sample was indented. The 60 s plateau time, higher than that found in the literature, was chosen from initial tests because wet tissue displays more viscous mechanical behavior. The unloading tangent was used to calculate the tissue elastic modulus from the indent load-displacement curve (Oliver & Pharr method^28^). For all samples, the Poisson’s ratio was assumed to be 0.3^29,30^. The STB tissue elastic moduli were obtained within 72 hours following surgery, with the samples kept hydrated throughout the process.

### Bone microarchitecture

Following indentation, the samples were removed from the sample holder and cleaned in the ultrasonic bath filled with soapy water for 15 min. Subsequently, they were defatted in a 1:1 methanol – diethylether solution for one day^31^. They were air-dried before being placed, dry and defatted, in the cone beam micro-CT (EasyTom XL Micro, RX Solutions, Chavanod, France). The cylindrical axis of each sample was aligned with the system’s rotation axis. The samples were scanned using fixed acquisition parameters: source voltage 118 kVp, current 81 µA, 1120 rotation steps (≈0.32°) over 360°, exposure time 330 ms, six-frame averaging, Field of View width 12.2 mm. Beam hardening was attenuated with a 0.4 mm-thick aluminum filter. For each sample, a 3D reconstruction window was positioned and slightly rotated to make the indented face perpendicular to the Z-axis of the image stack. The consecutive cross-section images were reconstructed using a filtered back-projection algorithm. Each image was saved in a 16 bit grayscale format (X-Act CT Software, V1.1, RX Solutions, Chavanod, France). Voxel sizes were constant accross samples at 8.4 × 8.4 × 8.4 µm^3^.

After reconstruction, all slices of all samples were visually checked. A subchondral trabecular bone volume of interest of 6 mm square base and variable height centered on the sample cylindrical axis was defined for each sample. In the proximal-distal direction, the VOI extended from the slice corresponding to the indentation surface, to the last slice before those containing subchondral bone plate tissue (see Fig. 1). Only VOIs over 1 mm in height were processed on BoneJ to determine the bone volume fraction (BV/TV) and the mean trabecular thickness (Tb.Th). Overall the VOIs boundaries were located at least 0.5 mm away from the surfaces cut with the surgical tools.

### Statistical analysis

#### STB nanoindentation vs. HKA and KL score

The effect of HKA and KL score on nanoindentation elastic modulus was evaluated via linear mixed models. As a previous study showed that bone tissue mineralisation is lower for regions closer to the subchondral surface^14^, the distance from the indented surface to the subchondral surface, henceforth referred as depth from subchondral surface (DepthSBSurf), was added to the regressors. The effect of within- and between-subject variables was analysed using a linear mixed model adjusted for gender, age and BMI, taking subject and sample side (medial or lateral) as random effects and sample side, HKA, DepthSBSurf and mean KL score as fixed effects. DepthSBSurf, the parameter most correlated with nanoindentation modulus, was forward-entered into multiple linear regression analysis, considering sample side, HKA interaction and KL mean score. Moreover, the mean indentation modulus of each sample was calculated and used to calculate the medial to lateral ratios of mean nanoindentation modulus for each subject (M:L nanoindentation modulus). The association between HKA and M:L nanoindentation modulus was assessed using a Pearson’s correlation test.

#### STB microarchitecture vs. HKA and KL score

The effect of HKA and KL score on microarchitecture, for each STB microarchitecture parameter, was evaluated using a mixed model ANCOVA with HKA and KL scores as independent variables and sample side as a within-subject factor. In addition, the medial to lateral ratio of BV/TV (M:L BV/TV) was calculated to adjust for within-subject factors and its association with HKA angle was assessed using a Pearson’s correlation test. The same procedure was applied to the medial to lateral ratio of Tb.Th (M:L Tb.Th).

#### STB nanoindentation vs. STB microarchitecture

To evaluate potential relationships between indent modulus and microarchitectural parameters, a hierarchical regression approach was used. BV/TV and Tb.Th were successively added to a linear mixed model with age, gender, BMI and DepthSBSurf as fixed effects, and subject and sample side as random effects. In addition, relationships between the M:L nanoindentation modulus ratios and M:L BV.TV and M:L Tb.Th were explored.

All tests were performed on **R**, with a significance level *p* < 0.05. Pearson’s correlation tests were used, or if data were not normally distributed, a Kendall’s Tau-b test was used. Residual plots of linear regression models were visually checked for assumptions of normality (Normal Q-Q plots), homogeneity of variance (scatter plots of residuals), linearity (residual vs fitted plots). For the figures, samples were stratified in two categories, “overloaded” or “underloaded”: the “overloaded” category applies to the medial sample in Varus subjects or the lateral sample in Valgus subjects, and conversely for the “underloaded” category.

### Ethics statement

As stipulated by the French Code of Research and the article L1245-2 of the Bioethic Law of 2011, the use of surgical waste doesn’t require formal approval by an ethic committee.

## Results

### Nanoindentation testing results

Overall, 2150 indentations out of the 2400 performed were successful. 225 failed due to errors in contact detection (either too soon or too late) and the penetration depth – force curves were not recorded for 25 indentations. Values for each sample from every subject are in Supplementary Material 1.

### Micro-CT acquisitions and microarchitecture parameters computing

All 60 samples were successfully micro-CT scanned. 44 samples presented a VOI with a height of over 1 mm, allowing the calculation of microarchitectural parameters (BV/TV, Tb.Th). This yielded 20 subjects with both medial and lateral measurements and 4 subjects with measurements for only one side. Calculated microarchitecture parameter values for each sample from every subject are in Supplementary Material 1.

### STB nanoindentation vs. HKA and KL score

**Figure 2:**
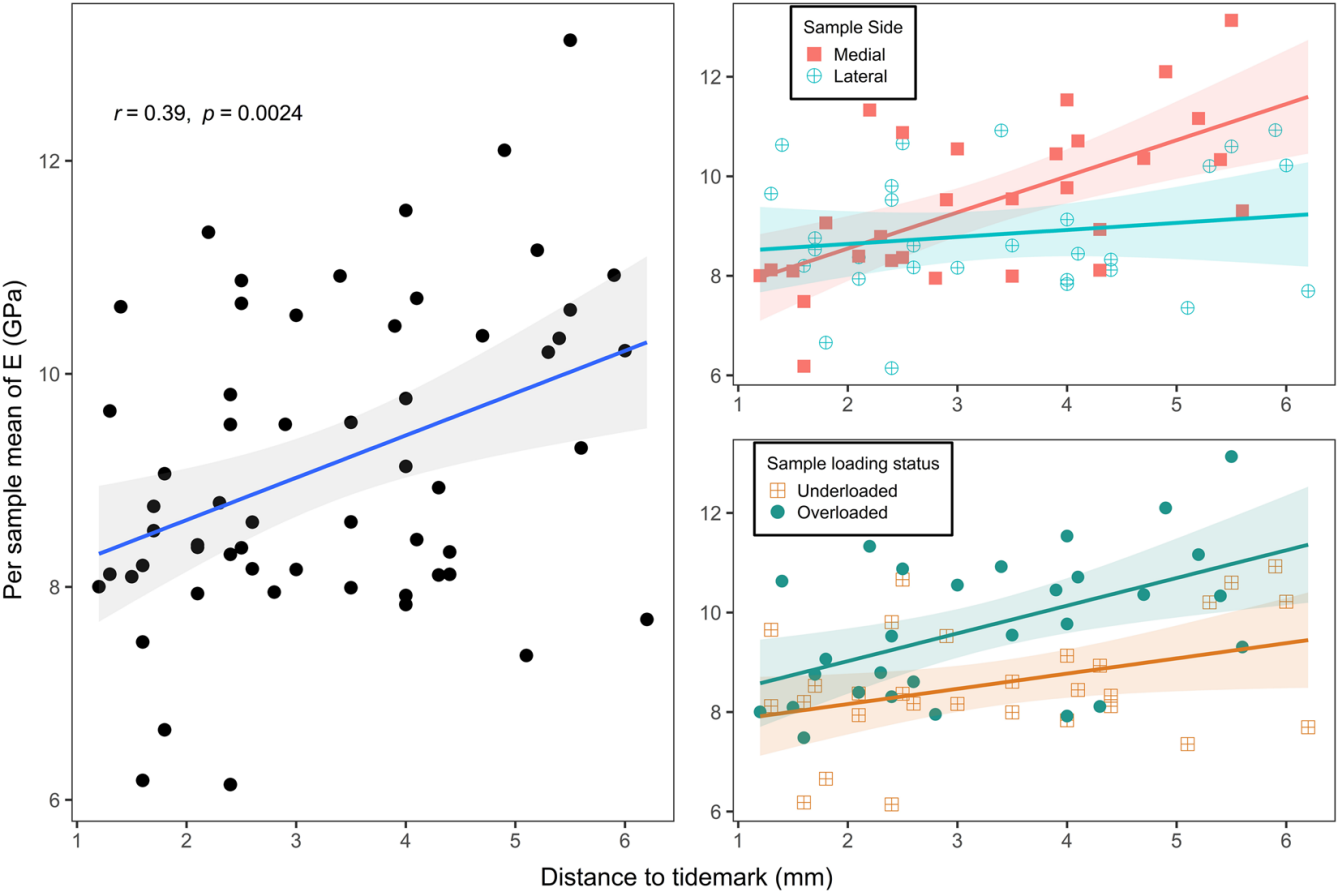
Scatter plots of the association between the depth from subchondral surface, namely the distance between the indented plane and the tidemark, and the per sample mean of E*, i.e., the mean nanoindentation elastic modulus of each subchondral bone sample. Best fit lines (solid lines) and 95% intervals (shaded area) for Pearson’s correlations: (left) all samples, (top right) samples stratified with sample side and (bottom right) samples stratified with loading status, with the medial samples of varus subjects considered overloaded and the lateral ones underloaded and vice-versa for the valgus subjects.

Visual inspection of residual plots did not reveal any obvious deviations from homoscedasticity, linearity or normality assumptions. However one subject was removed due to high leverage. Likelihood ratio tests of the model accounting for DepthSBSurf as a fixed effect vs. the null model showed DepthSBSurf to be a significant regressor (χ²(1) = 9.41 *P* = 0.0022, see Table 2). Sample mean nanoindentation moduli tended to increase with DepthSBSurf (R = 0.39 CI = [0.15, 0.59], p = 0.002, see Fig. 2).

**Table 2:** Summary of hierarchical linear regressions of indentation modulus vs clinical and microarchitectural parameters.

	Dependent variables	Models	Fixed Effects	Random Effects	χ²	*P*-value (>χ²)	*R*² (KR)	*R*² (SGV)
Hierarchical regression with clinical data	E*	mdl.0	Age, Gender, BMI	Subj/SS			0.04	0.01
	E*	mdl.1	mdl.0, DepthSBSurf	Subj/SS	9.41	0.002**	0.33	0.07
	E*	mdl.2	mdl.1, SS, SS:HKA	Subj/SS	10.09	0.018*.	0.43	0.11
	E*	mdl.3	mdl.2, SS:HKA:KL.Score	Subj/SS	9.96	0.007**	0.54	0.14
Hierarchical regression with microarchitectural data	E*	mdl.0	Age, Gender, BMI	Subj/SS			0.11	0.03
	E*	mdl.1	mdl.0, DepthSBSurf	Subj/SS	4.94	0.026*	0.30	0.06
	E*	mdl.2	mdl.1, BV.TV	Subj/SS	20.09	<0.001***	0.62	0.15
	E*	mdl.3	mdl.2, Tb.Th	Subj/SS	1.41	0.24	0.63	0.15
	E*	mdl.4	mdl.3, SS, SS:HKA	Subj/SS	0.92	0.82	0.63	0.16
	E*	mdl.5	mdl.4, SS:HKA:KL.Score	Subj/SS	1.36	0.51	0.65	0.16

Results of the two hierarchical linear regressions performed on the nanoindentation elastic modulus (E*) using mixed models to account for within-subject factors, null models (mdl.0) accounting for Age, Gender and, BMI. The first hierarchical regression investigated the link between clinical data and nanoindentation elastic modulus (E*). The second hierarchical regression investigated the link between microarchitectural parameters and nanoindentation elastic modulus (E*). Independent variables were successively added to the models’ fixed effects, and likelihood ratio tests were used between successive models to assess whether the added dependent variable affected the nanoindentation elastic modulus significantly. Likelihood ratio tests were performed with the Chi-Square test. “: “ In the models’ definition denotes the interaction between fixed effects. R² (KR) values here were calculated with the “ Kenward-Roger” approach, and R² (SGV) values were computed with “Standardised Generalised Variance”. When the name of a model is listed in the fixed effects, this model’s fixed effects are also the current model’s fixed effects. The following abbreviations were used in the model definitions: “DepthSBSurf” for Depth from subchondral surface; “SS” for Sample Side (medial or lateral); “BV.TV” for Bone volume fraction; “Tb.Th” for Trabecula thickness; “Subj” for Subjects; “HKA” for Hip-Knee-Ankle angle; “KL.Score” for per subject mean of measured Kellgren & Lawrence osteoarthritis scores. *p-value < 0.05, **p-value < 0.01, ***p-value < 0.001.

**Figure 3:**
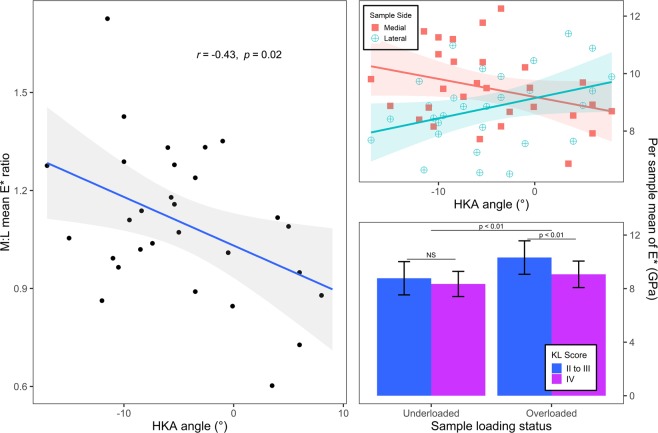
Plots illustrate relationships between per sample mean nanoindentation modulus (E*) and associated clinical parameters. The data were adjusted for depth from subchondral surface before plotting. For scatter plots, best fit lines (solid lines) and 95% intervals (shaded area) for Pearson’s correlations. (Left) Scatter plots of the association between the medial to lateral ratio of sample mean nanoindentation modulus (M:L mean E* ratio) and knee alignment (HKA angle). (top right) Scatter plots of per sample mean nanoindentation modulus (E*) and knee alignment (HKA angle). (bottom right) Bar plot of the per sample means of nanoindentation modulus (E*) stratified with both loading status and KL score. For KL score, subjects were separated into two groups: those rated IV by all surgeons and those not. Error bars indicate ±1 SD. For the bar plot, the horizontal lines indicate if the differences of the means are statistically significant according to Student’s t-tests.

The model using *sample side* and *HKA* as regressors improved the regression of the previous model (χ²(1) = 10.1 *P* = 0.018, see Table 2). Subsequently adding *sample side: HKA: mean KL score* interaction as a regressor further improved the previous model (χ²(2) = 9.96 *p* = 0.0069, see Table 2). M:L nanoindentation modulus was negatively correlated with HKA angle (R = −0.43 CI = [−0.69, −0.08], p = 0.019), meaning that the overloaded sides presented higher mean nanoindentation moduli than the underloaded sides. Moreover, moduli of overloaded samples were lower for KL IV subjects than for KL II to III subjects (see Fig. 3).

### STB microarchitecture vs. HKA and KL score

**Figure 4:**
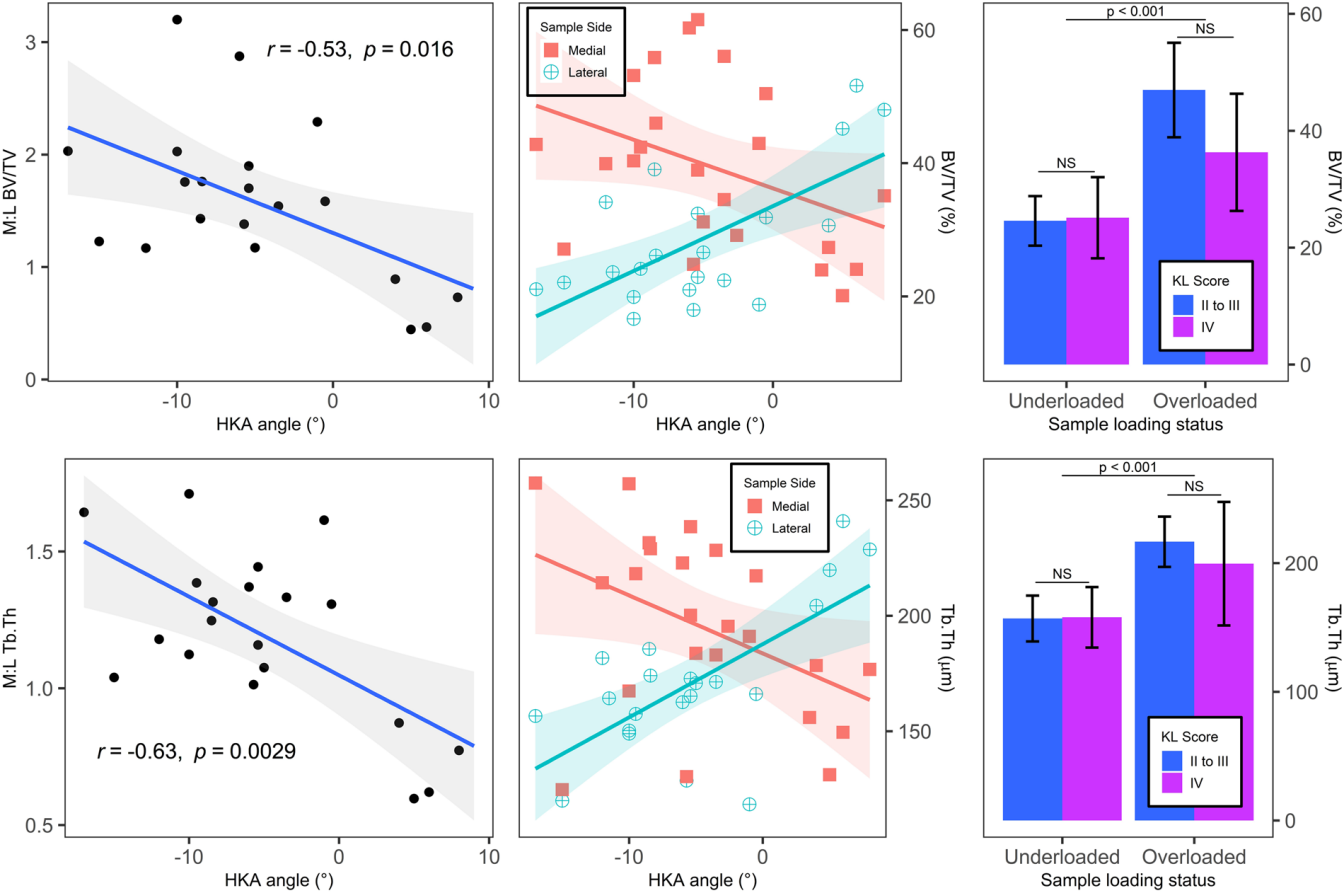
Plots illustrate relationships between microarchitectural parameters and associated clinical parameters. For scatter plots, data are presented along with best-fit lines (solid lines) and 95% intervals (shaded area) for Pearson’s correlations. For bar plots, the error bars denote ±1 SD. (Top left) Scatter plots of the association of the medial to lateral ratio of bone volume fraction (M:L BV/TV) with knee alignment (HKA angle). (Bottom left) Scatter plots of the association of the medial to lateral ratio of per sample mean trabecula thicknesses (M:L Tb.Th) with knee alignment (HKA angle). (Top middle) Scatter plots of per sample mean nanoindentation modulus (E*) and knee alignment (HKA angle).(top right) Bar plot of bone volume fraction (BV/TV) data stratified with both loading status and KL score. For KL score, subjects were separated into two groups: those rated IV by all surgeons and those not. Error bars indicate ±1 SD. (bottom right), the same as top right but for the per sample mean trabecula thickness (Tb.Th) data. For the bar plots, the horizontal lines indicate if the differences of the means are statistically significant according to Student’s t-tests.

For BV/TV, the analysis revealed an interaction between sample side and HKA angle (F(1, 15) = 26, 74, p < 0.001) and a triple interaction between sample side, HKA angle and mean KL score (F(1, 15) = 8.81, p = 0.0096). Moreover, M:L BV/TV displayed a moderate correlation with HKA angle (R = −0.53 CI = [−0.79, −0.11], p = 0.016). This translated into increasing BV/TV with increasing HKA for lateral samples, and inversely for medial samples. In other words, overloaded samples displayed higher BV/TV than underloaded samples within-subjects. This difference was reduced for KL grade IV subjects but not statistically significant (see Fig. 4).

The same interactions as for BV/TV were found significant for the analysis on Tb.Th (F(1, 15) = 30.21, p < 0.001 and F(1, 15) = 7.77, p = 0.0096, respectively). There was also a moderate correlation between M:L Tb.Th and HKA angle (R = −0.63 CI = [−0.84, −0.26], p = 0.003). Similarly to BV/TV, Tb.Th increased with increasing HKA angle for lateral samples and inversely for medial samples. The difference regarding KL grade IV was less marked, and not statistically significant, on Tb.Th (see Fig. 4).

### STB nanoindentation vs. STB microarchitecture

**Figure 5:**
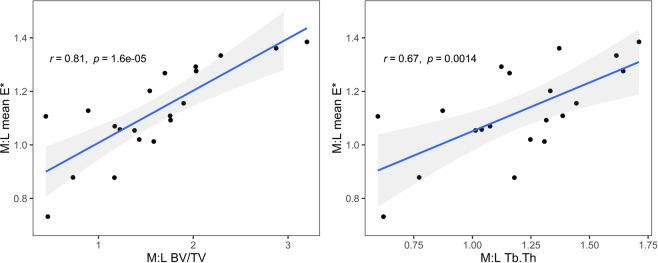
Scatter plots illustrate the association between nanoindentation modulus and microarchitectural parameters. Data are presented along with best-fit lines (solid lines) and 95% intervals (shaded area) for Pearson’s correlations. (left) Association between the medial to lateral ratio of sample mean nanoindentation modulus (M:L mean E*) and the medial to lateral ratio of bone volume fraction (M:L BV/TV). (right) Association between the medial to lateral ratio of sample mean nanoindentation modulus (M:L mean E*) and the medial to lateral ratio of per sample mean trabecula thicknesses (M:L Tb.Th).

Likelihood ratio tests of the model with BV/TV as a fixed effect vs. the model accounting only for depth from subchondral surface showed BV/TV to be a significant regressor (χ²(1) = 20.01 *P* < 0.001, see Table 2). Successively adding Tb.Th, sample side and HKA, and sample side: HKA: KL score interaction did not improve the regression (see Table 2). Medial:Lateral nanoindentation was strongly correlated with both Medial:Lateral Tb.Th (R = 0.67 CI = [0.32, 0.86], p = 0.0013) and Medial:Lateral BV/TV (R = 0.81 CI = [0.57, 0.92], p < 0.001), see Fig. 5.

## Discussion

This study examined the relationship between lower limb alignment, knee OA severity and tibial subchondral bone properties from samples surgically retrieved from patients undergoing TKA.

We used nanoindentation and micro-CT to measure the STB tissue elastic modulus and microarchitecture of medial and lateral STB samples of the proximal tibia from end-stage knee OA subjects. This allowed us to assess potential regional variations in micromechanical and microarchitectural properties of STB and to determine whether such regional variations are related to clinical data, i.e. lower limb malalignment and OA score. We also investigated relationships between the micromechanical and microarchitectural parameters.

These results should be interpreted in the light of the study’s limitations, which include the small number of subjects and the uneven proportions of men and women. However, the latter reflects the higher proportion of women undergoing TKA. In regressions, we used the mean KL score for each patient, calculated from 4 ratings by 2 raters, considering it as a continuous variable. This was done to enable discrimination between what were largely late-stage OA patients, by providing more resolution in the pathology stage assessment. Also, depending on limb alignment, we classified the medial and lateral tibial plateau samples as either overloaded or underloaded. However this classification accounted only for the intensity of the loading and not for its dynamics, which differ between the medial and lateral compartment. Under this classification, we found that the overloaded samples presented both larger quantities of STB and stiffer STB tissue than the underloaded samples. Moreoever, overloaded samples from subjects with very severe OA (KL score of 4) presented both lower quantities of STB and less tissue stiffness than less severe OA subjects (KL 3–4), although this was not true of underloaded samples.

Investigating the BMI effect on BV/TV, we found a weak and non-significant relationship between BV.TV and BMI for medial samples, whereas Reina *et al*. found a moderately significant relationship^32^. Like the Peters *et al*. study, we found no link between BMI and tissue elastic modulus^30^. These findings suggest that BMI should not have a major effect on STB tissue elastic modulus. It will take further studies with a larger, and possibly multicentric, cohort to confirm these results. In both cases, the lack of significance could be due to a lack of power: our sample size implies at least a 20% chance of not detecting correlation under 0.5.

We identified a statistically significant link between elastic modulus and HKA angle; the correlation was negative and moderate for medial regions and positive and weak for lateral regions. However, OA grade was also significantly associated with elastic modulus OA grade, and attenuated the correlation with HKA when OA severity increased. To the best of our knowledge, this study is the first to report a difference in STB elastic tissue moduli between overloaded and underloaded tibial condyles. This is consistent with a previous study showing a higher nanoindentation modulus on a sclerotic bone sample than on a relatively normal one from the same OA subject^33^. The STB elastic tissue moduli we measured are in the range of those measured by Wolfram *et al*. on rehydrated vertebrae trabecular bone^34^, and consistent with those measured on dehydrated tibial and femoral STB by Peters *et al*., allowing for the ~30% lower modulus for hydrated bone tissue^34^.

Peters *et al*. found no difference in trabecular bone elastic tissue modulus at different stages in the disease^30^. However their subjects presented less severe OA and their samples were tested dehydrated. As water content is higher in OA bone tissue^19^, its removal could reduce the differences between an OA and a normal tissue modulus. Also, our work identified a positive association between indentation modulus and distance from the subchondral surface to the indented face. Peters *et al*. did not report at what depth from the subchondral surface their indentation tests were performed; if it was very deep, the STB might not be noticeably affected by OA. This could also explain the absence of correlation with OA stage. An elastic modulus dependence on distance from the tidemark is consistent with the lower tissue mineralisation found in periarticular OA bone regions^19,35^. Proximal tibial OA samples present a gradient of mineralisation in STB, the tissue closer to the articular surfaces being less mineralised^14^. The STB mineralisation gradient is described as steeper for late-stage OA samples^14^. In line with these previous findings, we report a depth-dependent elastic modulus gradient, which was slightly steeper for overloaded samples.

We found that BV/TV and Tb.Th of the STB are significantly affected by HKA and OA stage: the bone volume fraction of the overloaded side can be three times that of the underloaded side. This is in line with previous studies showing higher STB BV/TV for the medial compartment of Varus OA subjects^12,16^. Our results confirm the previously described strong correlation between the medial to lateral ratio of BV/TV and mechanical axis deviation^17^. Mechanical axis deviation is highly correlated with HKA angle^36,37^. Trabecular thickness showed the same association. Our results also indicated that BV/TV is affected by OA stage, BV/TV being slightly lower on the overloaded side for severe OA patients (KL score of 4) than for moderate OA patients. In future studies, the investigation of the rod to plate ratio, as has been done for early OA^38^, could add another dimension to the analysis of the association of microarchitectural parameters with macroscopic loading and OA stage.

We highlighted a strong association between the Medial:Lateral elastic modulus ratio and the Medial:Lateral BV/TV ratio, within-subject ratios being used to reduce the influence of some subject-specific parameters (age, gender, BMI). This suggests that tissue micromechanical properties are linked to tissue quantity for late-stage OA, with greater bone quantity being associated with greater tissue-level stiffness. This is in line with the association between aBMD and nanoindentation modulus found by Kim *et al*. on osteoporotic vertebrae^39^.

The cross-sectional nature of this study prevented us from determining whether modifications to microarchitectural parameters alter the elastic modulus or vice-versa. Interplay among the parameters is also likely. Nor were we able to assess whether the relative reductions in modulus and BV/TV found in our very advanced OA subjects were directly due to the pathology or to pain-related disuse leading to physiological remodeling. Previous studies have shown that the ratio of trabecular bone macroscopic stiffness to bone volume fraction is reduced in OA sufferers^19^. We found that the overloaded side of the tibial plateau presented a higher tissue elastic modulus and higher BV/TV. The combination of these effects should lead to greater macroscopic stiffness^40^ of the overloaded STB compared to the underloaded STB.

Our observations indicate that the medial to lateral ratios of BV/TV and elastic moduli are related to HKA. This means that when there is a major malalignment, the bone stiffness on the underloaded side will be far less pronounced than on the overloaded side. This also suggests that macroscopic loading affects both micromechanical and microarchitectural properties of osteoarthritic STB. We explain this based on the following:

- In this study, the elastic modulus of the STB was measured in the central region of the trabeculae to avoid border effects.
- Overloaded samples present more bone (higher BV/TV) and larger trabeculae (higher Tb.Th) than the underloaded compartment.
- In advanced OA, there is a high STB bone turnover rate. As bone remodeling is a surface phenomenon, the newly deposited tissue will mainly be located on the outer surface of STB trabeculae.

The difference in STB tissue elastic modulus between the overloaded and underloaded compartments might not be due to a regional remodeling difference, but to the difference in trabecula thickness. The overloaded compartment presents larger trabeculae whose central part should be older, thus more mineralised and therefore stiffer, while the underloaded sample presents thinner trabeculae that should mainly be composed of recently deposited bone.

In this study, we demonstrated that the STB properties of late-stage OA subjects were related to routinely measured clinical parameters used to evaluate knee OA. We highlighted a regional variation of the mechanical properties of the STB tissue, and linked this variation with macroscopical loading. We also corroborated the previous finding of regional variation in microarchitectural STB parameters with loading^17^. Additionally, we found the medial to lateral ratios of STB quantity and STB tissue stiffness to be positively correlated; when inter-subject variations are evened out, the regional variations in STB quantity and tissue stiffness are strongly positively associated. Other studies, with possibly more subjects, are necessary to confirm those results and better explain the origins of the inter-subjects variations.

In late-stage OA, TKA is performed to alleviate pain and restore function. TKR implants are positioned to restore balanced loadings in the femorotibial compartments. A future study should investigate the properties of STB supporting the tibial implants plateau. If the alterations found here were present in the STB supporting the tibial implant, the pre-operatively underloaded compartment might be loaded by the implant beyond its reduced bearing capacity. This overloading could lead to bone resorption and fibrocartlilagenous tissue formation, resulting in tibial implant migration^41^, a major cause of TKA failure.

## Supplementary information

Supplementary Material 1.

## Data Availability

The microindentation and microarchitectural parameters dataset generated and analysed during the current study is available in the Open Science Framework repository, 10.17605/OSF.IO/EHF49. The μCT images datasets generated and analysed during the current study are available from the corresponding author on reasonable request.

## References

[1] Sharma L (2001). The Role of Knee Alignment in Disease Progression and Functional Decline in Knee Osteoarthritis. Jama.

[2] Brouwer GM (2007). Association between valgus and varus alignment and the development and progression of radiographic osteoarthritis of the knee. Arthritis Rheum..

[3] Miyazaki T, Wada M, Kawahara H (2002). Dynamic load at baseline can predict radiographic disease progression in medial compartment knee osteoarthritis. Ann. Rheum. Dis..

[4] Wada M (2001). Relationships among bone mineral densities, static alignment and dynamic load in patients with medial compartment knee osteoarthritis. Rheumatology.

[5] Hurwitz DE, Ryals AR, Karar A, Case JP, Andriacchi TP (2002). Static Alignment is a Better Indicator of the Dynamic Knee Joint Loads During Gait in Subjects with Knee Osteoarthritis Than Radiographic Disease Severity, Toe Out Angle and Pain. Orthop. Res. Soc..

[6] Rivière C (2017). Alignment options for total knee arthroplasty: A systematic review. Orthop. Traumatol. Surg. Res..

[7] Burr David B., Gallant Maxime A. (2012). Bone remodelling in osteoarthritis. Nature Reviews Rheumatology.

[8] Hunter DJ (2009). Bone marrow lesions from osteoarthritis knees are characterized by sclerotic bone that is less well mineralized. Arthritis Res. Ther..

[9] Kazakia GJ (2013). Bone and cartilage demonstrate changes localized to bone marrow edema-like lesions within osteoarthritic knees. Osteoarthr. Cartil..

[10] Tanamas SK (2010). Bone marrow lesions in people with knee osteoarthritis predict progression of disease and joint replacement: A longitudinal study. Rheumatology.

[11] Crema MD (2010). Subchondral Cystlike Lesions Develop Longitudinally in Areas of Bone Marrow Edema–like Lesions in Patients with or at Risk for Knee Osteoarthritis: Detection with MR Imaging—The MOST Study. Radiology.

[12] Roberts BC (2017). Systematic mapping of the subchondral bone 3D microarchitecture in the human tibial plateau: Variations with joint alignment. J. Orthop. Res..

[13] Finnilä MAJ (2017). Association between subchondral bone structure and osteoarthritis histopathological grade. J. Orthop. Res..

[14] Cox LGE, van Donkelaar CC, van Rietbergen B, Emans PJ, Ito K (2012). Decreased bone tissue mineralization can partly explain subchondral sclerosis observed in osteoarthritis. Bone.

[15] Patel V (2003). MicroCT evaluation of normal and osteoarthritic bone structure in human knee specimens. J. Orthropedic Res..

[16] Ding M, Odgaard A, Hvid I (2003). Changes in the three-dimensional microstructure of human tibial cancellous bone in early osteoarthritis. J. Bone Joint Surg. Br..

[17] Roberts BC (2018). Relationships between *in vivo* dynamic knee joint loading, static alignment and tibial subchondral bone microarchitecture in end-stage knee osteoarthritis. Osteoarthr. Cartil..

[18] Zysset PI, Sonny M, Hayes WC (1994). Morphology-mechanical Property Relations in Trabecular Bone of the Osteoarthritic proximal Tibia. J. Arthroplasty.

[19] Li B, Aspden RM (1997). Composition and mechanical properties of cancellous bone from the femoral head of patients with osteoporosis or osteoarthritis. J. Bone Miner. Res..

[20] Day JS (2001). A decreased subchondral trabecular bone tissue elastic modulus is associated with pre-arthritic cartilage damage. J. Orthop. Res..

[21] Ding M, Danielsen CC, Hvid I (2001). Bone density does not reflect mechanical properties in early-stage arthrosis. Acta Orthop. Scand..

[22] Mansell JP, Bailey AJ (1998). Abnormal cancellous bone collagen metabolism in osteoarthritis. J. Clin. Invest..

[23] Bailey AJ, Mansell JP, Sims TJ, Banse X (2004). Biochemical and mechanical properties of subchondral bone in osteoarthritis. Biorheology.

[24] Dall’Ara E, Öhman C, Baleani M, Viceconti M (2011). Reduced tissue hardness of trabecular bone is associated with severe osteoarthritis. J. Biomech..

[25] Coats AM, Zioupos P, Aspden RM (2003). Material properties of subchondral bone from patients with osteoporosis or osteoarthritis by microindentation testing and electron probe microanalysis. Calcif. Tissue Int..

[26] Gustafson MB (1996). Calcium buffering is required to maintain bone stiffness in saline solution. J. Biomech..

[27] Tawy GF, Rowe PJ, Riches PE (2016). Thermal Damage Done to Bone by Burring and Sawing With and Without Irrigation in Knee Arthroplasty. J. Arthroplasty.

[28] Oliver C, Pharr M (1992). An improved technique for determining hardness and elastic modulus using load and displacement sensing indentation experiments. Journal of Materials Research.

[29] Rodriguez-Florez N, Oyen ML, Shefelbine SJ (2013). Insight into differences in nanoindentation properties of bone. J. Mech. Behav. Biomed. Mater..

[30] Peters AE, Akhtar R, Comerford EJ, Bates KT (2018). The effect of ageing and osteoarthritis on the mechanical properties of cartilage and bone in the human knee joint. Sci. Rep..

[31] Cai X (2017). Cortical bone elasticity measured by resonant ultrasound spectroscopy is not altered by defatting and synchrotron X-ray imaging. J. Mech. Behav. Biomed. Mater..

[32] Reina N (2017). BMI-related microstructural changes in the tibial subchondral trabecular bone of patients with knee osteoarthritis. J. Orthop. Res..

[33] Zuo Q (2016). Characterization of nano-structural and nano-mechanical properties of osteoarthritic subchondral bone. BMC Musculoskelet. Disord..

[34] Wolfram U, Wilke HJ, Zysset PK (2010). Rehydration of vertebral trabecular bone: Influences on its anisotropy, its stiffness and the indentation work with a view to age, gender and vertebral level. Bone.

[35] Tomanik M, Nikodem A, Filipiak J (2016). Microhardness of human cancellous bone tissue in progressive hip osteoarthritis. J. Mech. Behav. Biomed. Mater..

[36] Paley Dror (2002). Principles of Deformity Correction.

[37] Bellemans J, Colyn W, Vandenneucker H, Victor J (2011). The Chitranjan Ranawat Award: Is Neutral Mechanical Alignment Normal for All Patients?: The Concept of Constitutional Varus. Clin. Orthop. Relat. Res..

[38] Chen Y (2018). Subchondral Trabecular Rod Loss and Plate Thickening in the Development of Osteoarthritis. J. Bone Miner. Res..

[39] Kim G, Cole JH, Boskey AL, Baker SP, Van Der Meulen MCH (2014). Reduced tissue-level stiffness and mineralization in osteoporotic cancellous bone. Calcif. Tissue Int..

[40] Wolfram U, Wilke HJ, Zysset PK (2010). Valid µ finite element models of vertebral trabecular bone can be obtained using tissue properties measured with nanoindentation under wet conditions. J. Biomech..

[41] Pijls BG, Plevier JWM, Nelissen RGHH (2018). RSA migration of total knee replacements. Acta Orthop..

