# Supplementary material for: Tibial subchondral trabecular bone micromechanical and microarchitectural properties are affected by alignment and osteoarthritis stage
Source: Sci Rep. 2020 Mar 4;10:3975. doi: 10.1038/s41598-020-60464-x (PMC7055326; doi:10.1038/s41598-020-60464-x)
Supplement: Supplementary file 1 — Supplementary Material 1. [file 41598_2020_60464_MOESM1_ESM.docx]

## Supplementary Material of :

# **Tibial subchondral trabecular bone micromechanical and microarchitectural properties are affected by alignment and osteoarthritis stage.**

Authors

Jean-Baptiste RENAULT^a,b,*^

Maximiliano CARMONA^b,c^

Chris TZIOUPIS^b^

Matthieu OLLIVIER^a,b^

Jean-Noël ARGENSON^a,b^

Sébastien PARRATTE^a,b,d^

Patrick CHABRAND^a,b^

* Corresponding author

a. Aix Marseille Univ, CNRS, ISM, Marseille, France

b. APHM, Institute for Locomotion, Department of orthopaedics and Traumatology, Sainte-Marguerite Hospital, 13009, Marseille, France

c. Department of Orthopaedics and Traumatology, Pontificia Universidad Católica de Chile, Santiago, Chile.

d. International Knee And Joint Center, Abu Dhabi, United Arabs Emirates.

**Power analysis**

We determine the number of subjects necessary using power analysis performed with “pwr” package in **R**. We fixed our wanted power at 0.80 and significance level at 0.05.

Roberts et al. study showed the Medial to Lateral ratio (M:L) of BV/TV to be correlated with the mechanical axis deviation with a coefficient of correlation of 0.74. So using a conservative approach we fixed our correlation effect to 0.6, it wielded to a minimum sample size of 19 subjects to evaluate the correlation between M:L of BV/TV and HKA angle.

To be able to evaluate if the medial and the lateral samples of each tibial plateau present different subchondral trabecular bone (STB) tissue elastic modulus we used the data present in the supplementary material of Peters et Al. (2018) study. We assumed a within sample standard deviation of 1.5 GPa, and wanted to be able to detect differences of 1 GPa between groups. This yield to a minimum of 36 indents per sample. Accounting for the 5% of indents that can be unexploitable when indenting trabecular bone Wolfram et Al. (2010), we chose to perform 40 indents per sample.

To be able to assess if the mean STB tissue elastic modulus of each sample were different depending on the overloaded/underloaded status of the sample. We wanted to be able to detect difference superior to 1 GPa assuming a standard deviation of 1.2 GPa as suggested by Peters et Al results for late stage STB tissue. This resulted in a minimum sample size of 26 subjects.

We pushed our number of subjects to 30 to be able to detect over 0.5 correlations between M:L of STB tissue elastic modulus and HKA angle.

|  |  |  | Kl Scores | | | | | | | | | |
| --- | --- | --- | --- | --- | --- | --- | --- | --- | --- | --- | --- | --- |
|  |  |  | Operator 1 | |  |  | Operator 2 | |  |  | Mean | Overall Grade |
|  |  |  | Evaluation 1 | Evaluation 2 |  |  | Evaluation 1 | Evaluation 2 |  |  |  |  |
|  |  |  |  |  |  |  |  |  |  |  |  |  |
| Subjects | E7F14AR |  | 4 | 3 |  |  | 4 | 4 |  |  | 3,75 | 4 |
|  | M9F13BL |  | 4 | 3 |  |  | 4 | 4 |  |  | 3,75 | 4 |
|  | E9M12BL |  | 4 | 4 |  |  | 4 | 4 |  |  | 4 | 4 |
|  | A10M13BL |  | 4 | 3 |  |  | 4 | 4 |  |  | 3,75 | 4 |
|  | N13F15BL |  | 4 | 3 |  |  | 4 | 4 |  |  | 3,75 | 4 |
|  | E14M13CR |  | 3 | 2 |  |  | 4 | 4 |  |  | 3,25 | 3 |
|  | A9F12CR |  | 3 | 3 |  |  | 3 | 3 |  |  | 3 | 3 |
|  | O9F17DR |  | 4 | 3 |  |  | 4 | 4 |  |  | 3,75 | 4 |
|  | M11F12DR |  | 4 | 3 |  |  | 4 | 4 |  |  | 3,75 | 4 |
|  | E12F13DL |  | 3 | 2 |  |  | 2 | 2 |  |  | 2,25 | 2 |
|  | R13M13FR |  | 4 | 3 |  |  | 4 | 4 |  |  | 3,75 | 4 |
|  | S14F16GR |  | 3 | 3 |  |  | 3 | 3 |  |  | 3 | 3 |
|  | N15F10GL |  | 4 | 3 |  |  | 4 | 4 |  |  | 3,75 | 4 |
|  | L14M10HR |  | 4 | 4 |  |  | 4 | 4 |  |  | 4 | 4 |
|  | I9F10JL |  | 4 | 4 |  |  | 4 | 4 |  |  | 4 | 4 |
|  | I11F16KL |  | 4 | 3 |  |  | 4 | 4 |  |  | 3,75 | 4 |
|  | S12F15MR |  | 3 | 2 |  |  | 2 | 2 |  |  | 2,25 | 2 |
|  | L8F12MR |  | 2 | 2 |  |  | 2 | 2 |  |  | 2 | 2 |
|  | G14F14MR |  | 3 | 3 |  |  | 3 | 3 |  |  | 3 | 3 |
|  | E6F15PR |  | 3 | 2 |  |  | 3 | 3 |  |  | 2,75 | 3 |
|  | R9M16PL |  | 4 | 2 |  |  | 4 | 3 |  |  | 3,25 | 3 |
|  | N8F12PR |  | 4 | 3 |  |  | 4 | 4 |  |  | 3,75 | 4 |
|  | N14M13PL |  | 4 | 3 |  |  | 4 | 4 |  |  | 3,75 | 4 |
|  | T10F14RL |  | 4 | 2 |  |  | 3 | 3 |  |  | 3 | 3 |
|  | N14F16RR |  | 4 | 3 |  |  | 4 | 4 |  |  | 3,75 | 4 |
|  | A8M15RR |  | 4 | 3 |  |  | 4 | 4 |  |  | 3,75 | 4 |
|  | G8F14RR |  | 4 | 3 |  |  | 4 | 4 |  |  | 3,75 | 4 |
|  | U10F11RR |  | 4 | 3 |  |  | 4 | 4 |  |  | 3,75 | 4 |
|  | S7F16TR |  | 4 | 4 |  |  | 4 | 4 |  |  | 4 | 4 |
|  | N9F18VR |  | 4 | 3 |  |  | 4 | 4 |  |  | 3,75 | 4 |

Table 1: Kellgren and Lawrence (KL) grading of all subjects blindly performed twice by two orthopedic surgeons.

|  |  | Age | Gender | HKA (°) | BMI |  | Nanoindentation Elastic Modulus (GPa) | | | | | | | |
| --- | --- | --- | --- | --- | --- | --- | --- | --- | --- | --- | --- | --- | --- | --- |
|  |  |  |  |  |  |  | Medial (Mean ± SD) | | |  |  | Lateral (Mean ± SD) | | |
|  | A10M13BL | 73 | M | -10.5 | 26.7 |  | 7.95 | ± | 2.09 |  |  | 8.17 | ± | 2.95 |
| Subjects | A8M15RR | 62 | M | -5.4 | 24.5 |  | 11.33 | ± | 2.54 |  |  | 9.81 | ± | 2.96 |
|  | A9F12CR | 72 | F | -5.4 | 29.0 |  | 10.71 | ± | 1.98 |  |  | 8.44 | ± | 1.90 |
|  | E14M13CR | 77 | M | -3.5 | 35.6 |  | 7.48 | ± | 1.91 |  |  | 8.53 | ± | 2.01 |
|  | E6F15PR | 60 | F | 4.2 | 22.7 |  | 4.49 | ± | 0.87 |  |  | 9.76 | ± | 1.32 |
|  | E7F14AR | 61 | F | -5.7 | 29.1 |  | 8.11 | ± | 2.25 |  |  | 7.69 | ± | 1.83 |
|  | G14F14MR | 67 | F | 5.0 | 23.1 |  | 9.53 | ± | 2.32 |  |  | 8.61 | ± | 1.94 |
|  | G8F14RR | 71 | F | 6.0 | 30.5 |  | 8.12 | ± | 1.72 |  |  | 8.76 | ± | 2.23 |
|  | I11F16KL | 83 | F | -2.6 | 24.2 |  | 8.31 | ± | 1.76 |  |  | 6.14 | ± | 1.63 |
|  | I9F10JL | 72 | F | 8.0 | 28.0 |  | 8.37 | ± | 2.14 |  |  | 9.53 | ± | 2.72 |
|  | L12F11NL | 75 | F | -10.0 | 24.4 |  | 10.55 | ± | 2.76 |  |  | 8.16 | ± | 2.24 |
|  | L14M10HR | 68 | M | -12.0 | 29.4 |  | 9.30 | ± | 3.08 |  |  | 10.6 | ± | 2.45 |
|  | L8F12MR | 80 | F | 3.5 | 18.8 |  | 6.18 | ± | 1.88 |  |  | 10.63 | ± | 2.57 |
|  | M9F13BL | 72 | F | -15.0 | 25.6 |  | 8.39 | ± | 1.85 |  |  | 7.94 | ± | 1.88 |
|  | N10F17AR | 64 | F | -8.5 | 26.2 |  | 10.88 | ± | 2.73 |  |  | 10.66 | ± | 2.00 |
|  | N13F15BL | 67 | F | 4.0 | 28.7 |  | 8.93 | ± | 2.78 |  |  | 7.92 | ± | 1.73 |
|  | N14F16RR | 77 | F | -11.5 | 32.5 |  | 12.10 | ± | 2.41 |  |  | 7.36 | ± | 2.06 |
|  | N14M13PL | 68 | M | -7.4 | 27.7 |  | 8.79 | ± | 2.38 |  |  | 8.37 | ± | 1.67 |
|  | N15F10GL | 78 | F | 0.2 | 24.9 |  | 8.00 | ± | 1.78 |  |  | 9.65 | ± | 1.41 |
|  | N8F12PR | 71 | F | -5.0 | 28.6 |  | 9.77 | ± | 2.20 |  |  | 9.13 | ± | 1.86 |
|  | N9F18VR | 72 | F | 0.5 | 28.2 |  | 10.33 | ± | 2.16 |  |  | 10.2 | ± | 2.30 |
|  | O9F17DR | 72 | F | -9.5 | 37.1 |  | 9.55 | ± | 2.40 |  |  | 8.61 | ± | 2.57 |
|  | R13M13FR | 76 | M | -11.0 | 29.4 |  | 8.10 | ± | 1.90 |  |  | 8.20 | ± | 1.36 |
|  | R9M16PL | 72 | M | -8.4 | 27.9 |  | 11.16 | ± | 3.04 |  |  | 10.22 | ± | 2.25 |
|  | S12F15MR | 75 | F | -1.0 | 35.3 |  | 10.45 | ± | 2.11 |  |  | 7.83 | ± | 2.60 |
|  | S14F16GR | 68 | F | 6.0 | 28.3 |  | 7.99 | ± | 1.77 |  |  | 10.92 | ± | 2.25 |
|  | S7F16TR | 70 | F | -17.0 | 27.1 |  | 10.36 | ± | 2.74 |  |  | 8.12 | ± | 2.57 |
|  | T10F14RL | 82 | F | -3.5 | 31.2 |  | 13.13 | ± | 3.74 |  |  | 10.93 | ± | 2.38 |
|  | T12F14RL | 84 | F | -6.0 | 28.9 |  | 9.06 | ± | 2.18 |  |  | 6.66 | ± | 1.40 |
|  | U10F11RR | 73 | F | -10.0 | 43.3 |  | 11.53 | ± | 2.47 |  |  | 8.33 | ± | 1.94 |

Table 2: Nanoindentation elastic modulus of each subjects’ medial and lateral samples.

|  |  | BV/TV (%) | |  | Tb.Th (μm) | | | | | | |
| --- | --- | --- | --- | --- | --- | --- | --- | --- | --- | --- | --- |
|  |  | Medial | Lateral |  | Medial (Mean ± SD) | | |  | Lateral (Mean ± SD) | | |
|  | A10M13BL | *NA* | *NA* |  | *NA* | | |  | *NA* | | |
| Subjects | A8M15RR | 61.5 | 32.4 |  | 239 | ± | 69 |  | 165 | ± | 49 |
|  | A9F12CR | 38.9 | 22.9 |  | 200 | ± | 57 |  | 173 | ± | 58 |
|  | E14M13CR | 56.0 | *NA* |  | 183 | ± | 73 |  | *NA* | | |
|  | E6F15PR | *NA* | *NA* |  | *NA* | | |  | *NA* | | |
|  | E7F14AR | 24.8 | 18.0 |  | 130 | ± | 44 |  | 129 | ± | 52 |
|  | G14F14MR | 20.1 | 45.2 |  | 131 | ± | 52 |  | 220 | ± | 85 |
|  | G8F14RR | *NA* | *NA* |  | *NA* | | |  | *NA* | | |
|  | I11F16KL | 29.1 | *NA* |  | 195 | ± | 70 |  | *NA* | | |
|  | I9F10JL | 35.1 | 48.0 |  | 177 | ± | 59 |  | 229 | ± | 71 |
|  | L12F11NL | 40.3 | 19.9 |  | 167 | ± | 51 |  | 149 | ± | 60 |
|  | L14M10HR | 39.9 | 34.2 |  | 214 | ± | 63 |  | 182 | ± | 59 |
|  | L8F12MR | 24.0 | *NA* |  | 156 | ± | 62 |  | *NA* | | |
|  | M9F13BL | 27.1 | 22.1 |  | 125 | ± | 40 |  | 120 | ± | 45 |
|  | N10F17AR | 55.9 | 39.1 |  | 232 | ± | 75 |  | 186 | ± | 51 |
|  | N13F15BL | 27.3 | 30.6 |  | 178 | ± | 62 |  | 204 | ± | 87 |
|  | N14F16RR | *NA* | 23.6 |  | *NA* | | |  | 164 | ± | 63 |
|  | N14M13PL | *NA* | *NA* |  | *NA* | | |  | *NA* | | |
|  | N15F10GL | *NA* | *NA* |  | *NA* | | |  | *NA* | | |
|  | N8F12PR | 31.2 | 26.6 |  | 184 | ± | 57 |  | 171 | ± | 64 |
|  | N9F18VR | 50.4 | 31.9 |  | 217 | ± | 67 |  | 166 | ± | 55 |
|  | O9F17DR | 42.4 | 24.1 |  | 218 | ± | 68 |  | 157 | ± | 58 |
|  | R13M13FR | *NA* | *NA* |  | *NA* | | |  | *NA* | | |
|  | R9M16PL | 46.0 | 26.1 |  | 229 | ± | 78 |  | 174 | ± | 55 |
|  | S12F15MR | 43.0 | 18.8 |  | 191 | ± | 58 |  | 118 | ± | 41 |
|  | S14F16GR | 24.0 | 51.6 |  | 150 | ± | 44 |  | 241 | ± | 68 |
|  | S7F16TR | 42.8 | 21.1 |  | 257 | ± | 98 |  | 157 | ± | 54 |
|  | T10F14RL | 34.5 | 22.4 |  | 228 | ± | 74 |  | 171 | ± | 58 |
|  | T12F14RL | 60.3 | 21.0 |  | 223 | ± | 71 |  | 163 | ± | 61 |
|  | U10F11RR | 53.2 | 16.6 |  | 257 | ± | 70 |  | 150 | ± | 58 |

Table 3: Microarchitectural parameters for every subject. Samples not presenting enough subchondral trabecular bone volume for correct evaluation of the parameters from the microcomputed tomography reconstructions, were not evaluated and noted as “NA”. BV/TV: Bone volume fraction. Tb.Th: Trabeculae thickness.
